# Use of social adaptability index to explain self-care and diabetes outcomes

**DOI:** 10.1186/s12902-017-0185-3

**Published:** 2017-06-20

**Authors:** Jennifer A. Campbell, Rebekah J. Walker, Brittany L. Smalls, Leonard E. Egede

**Affiliations:** 1 Center for Patient Care and Outcomes Research (PCOR), Medical College of Wisconsin, 8701 Watertown Plank Road, Milwaukee, WI 53226 USA; 2 Division of General Internal Medicine, Medical College of Wisconsin, Milwaukee, WI USA; 3 Center for Health Services Research, University of Kentucky, Lexington, KY USA

**Keywords:** Social Adaptability Index, Diabetes, Self-care, Glycemic control

## Abstract

**Background:**

To examine whether the social adaptability index (SAI) alone or components of the index provide a better explanatory model for self-care and diabetes outcomes.

**Methods:**

Six hundred fifteen patients were recruited from two primary care settings. A series of multiple linear regression models were run to assess (1) associations between the SAI and diabetes self-care/outcomes, and (2) associations between individual SAI indicator variables and diabetes self-care/outcomes. Separate models were run for each self-care behavior and outcome. Two models were run for each dependent variable to compare associations with the SAI and components of the index.

**Results:**

The SAI has a significant association with the mental component of quality of life (0.23, *p* < 0.01). In adjusted analyses, the SAI score did not have a significant association with any of the self-care behaviors. Individual components from the index had significant associations between self-care and multiple SAI indicator variables. Significant associations also exist between outcomes and the individual SAI indicators for education and employment.

**Conclusions:**

In this population, the SAI has low explanatory power and few significant associations with diabetes self-care/outcomes. While the use of a composite index to predict outcomes within a diabetes population would have high utility, particularly for clinical settings, this SAI lacks statistical and clinical significance in a representative diabetes population. Based on these results, the index does not provide a good model fit and masks the relationship of individual components to diabetes self-care and outcomes. These findings suggest that five items alone are not adequate to explain or predict outcomes for patients with type 2 diabetes.

## Background

Diabetes is the seventh leading causes of death among adults in the US population and affects more than 382 billion people worldwide [1, 2]. It is associated with an increased risk of mortality, heart disease, and stroke, and is the leading cause of kidney failure [1]. Each year, diabetes expenditures in the US reach more than 160 billion dollars in direct and indirect medical costs, and those living with diabetes have two times the medical expenditures as those living without it [1]. Additionally, in 2012 diabetes was responsible for an estimated 1.5 million deaths worldwide [2]. Recent work on the social determinants of health found that diabetes outcomes are influenced by factors such as level of education, economic conditions, and social support with specific factors being found to have a direct relationship with diabetes self-care behaviors [3–9]. As such, the need for tailored interventions and treatment plans that target individual, social, and behavioral factors associated with diabetes care are being given greater attention [10–12]. However, better understanding of the underlying mechanisms that increase risk of poor diabetes outcomes, outside of traditional demographic factors, may lend to greater methods of prevention and development of treatment interventions.

The newly developed Social Adaptability Index (SAI) is a composite indicator developed to be a more sensitive and specific mechanism to capture both an individual’s social adaptability within society and socioeconomic status in order to predict overall health risk [13–16]. Based on factors including employment status, substance abuse, marital status, and income, the SAI provides a composite score that has been correlated with health outcomes and overall mortality [13]. Primarily tested in patients with chronic kidney disease, the SAI was found to be a predictor of mortality due to chronic kidney disease, and has since been used as a predictor in overall mortality, depression, as well as access to kidney transplantation [13–16]. More recently, the SAI was found to be a predictor of mortality in patients with diabetes through a dose response relationship between SAI score and mortality in US adults [13–16].

While the current literature supports the SAI as a predictor of kidney transplant outcomes as well as mortality in chronic kidney disease and diabetes, little has been done to determine the relationship between SAI, self-care, and diabetes outcomes. Previous work noted that by using a composite index rather than traditional demographic criteria for identifying populations at risk for poor health outcomes, SAI has the potential to be more sensitive and specific [15, 16]. Therefore, the aim of this analysis was to evaluate the SAI in a population of patients with type 2 diabetes. We examined whether the index alone or components of the index provide a better explanatory model for self-care and diabetes outcomes. Based on the literature, we hypothesized that SAI would have a significant and negative association with diabetes self care and outcomes, including glycemic control, blood pressure, lipids, and quality of life in patients with type 2 diabetes, and would show a stronger association than the individual components.

## Methods

### Study population

A convenience sample of participants were recruited from two primary care clinics in the southeastern United States from the state of South Carolina. Research coordinators cross-checked the primary care clinic patient pool with electronic medical records to determine patient eligibility. Patients were eligible for the study if they were: age 18 years or older, diagnosed with type 2 diabetes, and were able to communicate in English. Patients were ineligible if cognitive impairment due to dementia or active psychosis was determined by chart documentation or interaction. Potential study participants were sent recruitment letters and those interested scheduled appointments to complete the survey with a research coordinator. All letters were approved by the local institutional review board. Additionally, patients within the primary care clinics were approached before or after their scheduled clinic visits and were assessed for eligibility and interest in study participation. Less than 10% of participants who were eligible for the study did not participate. Research coordinators explained study procedures to those who were interested in participating in the study. Participants were either consented during their current clinic visit, at their next clinic visit or at a scheduled study visit with a research coordinator. Following completion of the survey, research coordinators reviewed each study questionnaire for missing data and requested that patients complete any missing forms. A total of 60 patients had one or more missing variables at study completion.

At the time of consent, participants completed validated questionnaires with information on demographics, social determinants of health factors, self-care behaviors, and comorbidities. Social determinants included in the questionnaire were selected based on a modified version of the conceptual framework by Brown et al., elucidating pathways linking social determinants of health with outcomes in patients with type 2 diabetes [17]. Clinical outcomes, including HbA1c, blood pressure, and cholesterol (LDL) were abstracted from the medical record. The local institutional review board approved all study procedures prior to study recruitment and enrollment.

### Demographic covariates

Previously validated items from the 2002 National Health Interview Survey (NCHS 2004) were used to collect general demographic and socioeconomic information, including age, race, gender, years of education, marital status, income, and employment status. Duration of diabetes diagnosis and health status, scored on a scale of 1 to 5 with 1 being low and 5 being high, was self-reported by patients [18]. Medical comorbidity was collected and calculated using the Charlson comorbidity index [19].

### Social Adaptability Index (SAI) variables

The primary variables of interest were created from previously validated questions scored according to instructions developed using data in the US general population [14–16]. All variables chosen and scoring used matched previously developed SAI index. [14–16] The components of the social adaptability index (SAI) include: education level, employment status, income, marital status and substance abuse. Education, employment, and substance abuse were graded on a scale of 0 to 3, and marital status and income were graded on a scale of 0 to 2.

- Education level: 0 = no high school graduation (0–11 years of school), 1 = high school graduate (12 years of school), 2 = at least some college (13–16 years of school), 3 = post-college education (17–24 years of school).
- Employment status: 0 = unemployed and unable to work because of health, and not working by choice (student or homemaker), 1 = retired and not working, 2 = working part-time, 3 = working full time.
- Income: 0 = less than $20,000 per year per household, 1 = $20,000 - $50,000 per year per household, 2 = more than $50,000 per year per household.
- Marital status: 0 = not married (never married or widowed), 1 = separated or divorced, 2 = married. The original SAI analysis added 3 = married with children, but this information was not available in this dataset so we used a scale of only 0 to 2.
- Substance abuse: 0 = abusing drugs/alcohol and currently using tobacco, 1 = abusing drugs/alcohol, 2 = currently using tobacco, 3 = none. Use of drugs and alcohol was determined by answer to the CAGE questionnaire to measure drug and alcohol addiction [20]. Tobacco use was determined by answers to the question of whether a patient never smoked, was a former smoker or was a current smoker.

The SAI was calculated by adding all five factors for each participant, leading to a final scale with a range of 0 to 13. Lower scores indicated higher risk. Variables were also added individually to separate regression models and kept the same scoring as noted above.

### Self-care variables

Diabetes behavioral skills were assessed using the Summary of Diabetes Self-care Activities (SDSCA): an 11-item scale measuring the frequency of conducting self-care activities in the last 7 days. Activities included were general diet (following a healthy diet), specific diet (eating two fruits and two fat diet), exercise, blood glucose testing, and foot care [21].

### Outcomes

Quality of life was assessed using the SF-12: a 12-item scale providing a summary physical health (PCS-12) and mental health (MCS-12) component outcome scores. The SF-12 is a valid and reliable instrument (alpha = 0.89) [22, 23]. Hemoglobin A1c, blood pressure, and cholesterol (LDL) were abstracted from the medical record using the most recent values relative to the date of completed survey within the past 6 months for A1c and blood pressure, and within the past 12 months for LDL.

### Statistical analyses

After testing for normal distribution, means and percentages for all variables were calculated, including the SAI and the individual SAI components. A series of multiple linear regression models were run to assess (1) associations between the SAI and self-care and outcomes, and (2) associations between individual SAI indicator variables and self-care and outcomes. Separate models were run for each self-care behavior (general diet, specific diet, exercise, blood sugar testing, and foot care), and outcome (PCS, MCS, A1c, blood pressure, and LDL-lipids) as the dependent variable. Two models were run for each dependent variable: first with the SAI score as the independent variable, and second with the SAI indicator variables as multiple independent variables. Unadjusted analyses were conducted first, followed by models adjusted for age, race, gender, site, diabetes duration, health status and comorbidity. A two-tailed alpha of 0.05 was used to asses statistical significance and R^2^ value were used to assess model fit and explanatory power. Hypotheses for SAI index overall, and individual components of SAI to understand the utility of this index were set a priori, and therefore, statistical significance was not adjusted for multiple comparisons. All analyses were performed using Stata Version 13.

## Results

Patient characteristics for this sample of 615 adults with type 2 diabetes are summarized in Table 1. The majority of the population were non-Hispanic Blacks (64.9%) and men (61.6%), with a mean age of 61 years, mean education of 13.4 years, and a mean of 12.5 h worked per week. Participants had been diagnosed with type 2 diabetes for an average of 12.3 years.

**Table 1:** Sample demographic characteristics (*n* = 615)

	% or Mean ± standard deviation
Age (years)	61.3 ± 10.9
Education (years)	13.4 ± 2.8
Employment (hours worked per week)	12.5 ± 18.9
Diabetes Duration (years)	12.3 ± 9.1
Comorbidity (Charlson score)	25.7 ± 2.2
Health Status (score)	3.4 ± 0.9
Gender	
Women	38.4
Men	61.6
Race/Ethnicity	
Non-Hispanic Black	64.9
Non-Hispanic Whites	33.0
Hispanic/Other	2.1
Marital Status	
Never Married	11.2
Married	49.7
Separated/Divorced	28.2
Widowed	10.9
Site of Care	
Non-VAMC	51.2
VAMC	48.8
Annual income level	
< $10,000	20.2
$10,000–$14,999	11.3
$15,000–$19,999	10.1
$20,000–$24,999	10.4
$25,000–$34,999	14.7
$35,000–$49,999	13.8
$50,000–$74,999	10.1
$75,000+	9.4
HbA1c	7.9 ± 1.8
Self-care Behaviors	
General Diet	4.7 ± 2.0
Specific Diet	4.0 ± 1.5
Exercise	2.6 ± 2.2
Blood Sugar Testing	4.6 ± 2.5
Foot Care	4.3 ± 2.5

Tables 2 and 3 show the association between self-care behaviors and SAI. In the unadjusted models (Table 2), when SAI indicator variables were added individually, we found that specific diet had significant associations with employment (being retired: beta 0.43, *p* < 0.05) and substance abuse (no drug or alcohol use: beta 0.52, *p* < 0.05). Exercise showed a significant relationship with education (college education: beta 0.60, *p* < 0.05 and post-college education: beta 1.34, *p* < 0.01). There was a significant association between foot care and substance abuse (no drug or alcohol abuse: beta 0.85, *p* < 0.05).

**Table 2:** Unadjusted models for the relationship with self-care behaviors

	General Diet	Specific Diet	Exercise	Blood Sugar Test	Foot Care
Unadjusted Analyses					
SAI score	0.03	0.05	0.02	−0.03	−0.03
R^2^	0.0016	0.0063	0.0004	0.0008	0.0013
SAI indicator - education					
No high school degree (ref)	---	---	---	---	---
High school graduate	−0.34	−0.23	0.60	0.33	−0.02
College graduate	−0.31	−0.22	0.60*	0.40	−0.27
Post college	−0.01	−0.10	1.34**	−0.13	−0.05
SAI indicator - employment					
Unemployed (ref)	---	---	---	---	---
Retired	0.30	0.43*	0.05	−0.05	0.12
Working part time	−0.09	0.32	−0.06	−0.42	−0.23
Working full time	−0.16	0.12	0.43	−0.19	−0.09
SAI indicator – marital status					
Never married (ref)	---	---	---	---	---
Divorced/separated	0.19	0.10	0.22	−0.39	0.12
Married	0.04	0.12	0.16	0.04	0.04
SAI indicator – substance abuse					
Abusing drugs and alcohol (ref)	---	---	---	---	---
Abusing drugs	0.07	0.34	−0.07	−0.25	0.11
Abusing tobacco	0.38	0.41	−0.46	0.41	0.16
No drug or alcohol abuse	0.38	0.52*	−0.03	0.33	0.85*
SAI indicator – income					
> $20,000 (ref)	---	---	---	---	---
$20,000 - $49,999	0.16	−0.19	−0.86***	−0.36	−0.39
$50,000 and more	0.17	0.02	−0.55	−0.57	−0.79
R^2^	0.0208	0.0338	0.0471	0.0249	0.0354

B﻿old﻿﻿ ﻿= statistical significance, *p < 0.05, **p < 0.01, ***p < 0.001, ref. = reference A1c = hemoglobin A1c, PCS = physical component of quality of life, MCS = mental component of quality of life adjusted for age, race, gender, site, diabetes duration, health status and comorbidity

**Table 3:** Adjusted models for the relationship with self-care behaviors

	General Diet	Specific Diet	Exercise	Blood Sugar Test	Foot Care
Adjusted Analyses					
SAI score	0.02	0.04	−0.01	0.0009	0.02
R^2^	0.0949	0.0878	0.0470	0.0490	0.0854
SAI indicator - education					
No high school degree (ref)	---	---	---	---	---
High school graduate	−0.22	−0.07	0.56	0.56	0.19
College graduate	−0.07	0.04	0.64*	0.66	0.01
Post college	0.09	−0.01	1.39**	−0.10	0.16
SAI indicator - employment					
Unemployed (ref)	---	---	---	---	---
Retired	−0.06	0.19	−0.23	−0.21	−0.12
Working part time	−0.23	0.25	−0.25	0.002	0.005
Working full time	−0.11	0.11	0.24	0.14	0.17
SAI indicator – marital status					
Never married (ref)	---	---	---	---	---
Divorced/separated	0.28	0.22	0.29	−0.41	−0.15
Married	0.17	0.22	0.10	−0.05	0.11
SAI indicator – substance abuse					
Abusing drugs and alcohol (ref)	---	---	---	---	---
Abusing drugs	0.04	0.20	0.02	−0.33	0.20
Abusing tobacco	0.31	0.29	−0.30	0.19	0.28
No drug or alcohol abuse	0.17	0.24	0.13	0.10	0.86*
SAI indicator – income					
> $20,000 (ref)	---	---	---	---	---
$20,000 - $49,999	0.05	−0.12	−1.01***	−0.27	−0.44
$50,000 and more	0.09	0.08	−0.68*	−0.50	−0.61
R^2^	0.1007	0.0952	0.1042	0.0738	0.1058

Bold = statistical significance, *p < 0.05, **p < 0.01, ***p < 0.001, ref. = reference A1c = hemoglobin A1c, PCS = physical component of quality of life, MCS = mental component of quality of life adjusted for age, race, gender, site, diabetes duration, health status and comorbidity

In the adjusted model (Table 3), the SAI score did not have a significant association with any of the self-care behaviors. However, there were significant associations between self-care and individual SAI indicator variables. Exercise had a significant association with education (college education: beta 0.64, *p* < 0.05 and post-college education: beta 1.39, *p* < 0.01) as well as annual income ($20,000–$49,999: beta −1.01, *p* < 0.001 and >$50,000: beta −0.68, *p* < 0.05). Foot care showed a significant relationship with substance abuse (no drug or alcohol abuse: beta 0.86, *p* < 0.05).

Associations between health outcomes and quality of life and social adaptability are shown in Tables 4 and 5. The unadjusted model (Table 4) shows that HbA1c was significantly associated with employment (working fulltime: beta 0.66, *p* < 0.01) and substance abuse (abusing drugs: beta −0.65, *p* < 0.05, abusing alcohol: beta −0.74, *p* < 0.05, and no drug or alcohol abuse: beta 0.61, *p* < 0.05). Blood pressure had significant relationships with education (high school graduate: beta −5.15, *p* < 0.05 and college graduate: beta −6.12, *p* < 0.01) and substance abuse (no drug or alcohol abuse: beta 5.60, *p* < 0.05). MCS had significant associations with education (high school education: beta 1.07, *p* < 0.01 and post college education: beta 0.98, *p* < 0.05), employment (being retired: beta 0.70, *p* < 0.01, working part-time: beta 0.92, *p* < 0.05, and working fulltime: beta 1.16, *p* < 0.001). Lastly, lipids had a significant relationship with employment (being retired: beta −14.78, *p* < 0.05).

**Table 4:** Unadjusted models for the relationship with glycemic control and quality of life

	A1c	Blood Pressure	Lipids	PCS	MCS
Unadjusted Analyses					
SAI score	−0.19	0.24	−0.20	0.004	0.23***
R^2^	0.0008	0.0015	0.0001	0.0002	0.0584
SAI indicator - education					
No high school degree (ref)	---	---	---	---	---
High school graduate	−0.34	−5.15*	3.39	−0.19	1.07**
College graduate	−0.16	−6.12**	−2.11	−0.04	0.58
Post college	−0.62	−1.24	8.35	0.01	0.96*
SAI indicator - employment					
Unemployed (ref)	---	---	---	---	---
Retired	−0.03	−0.67	−14.78*	0.09	0.70**
Working part time	−0.26	0.64	0.75	0.28	0.92*
Working full time	0.66**	0.62	4.29	0.04	1.16***
SAI indicator – marital status					
Never married (ref)	---	---	---	---	---
Divorced/separated	0.27	0.44	−3.02	−0.01	−0.40
Married	0.33	−1.70	−5.69	−0.20	0.22
SAI indicator – substance abuse					
Abusing drugs and alcohol (ref)	---	---	---	---	---
Abusing drugs	−0.65*	3.75	5.19	0.10	−0.46
Abusing alcohol	−0.74*	2.79	−1.30	0.03	−0.01
No drug or alcohol abuse	−0.61*	5.60*	−3.34	0.03	0.26
SAI indicator – income					
> $20,000 (ref)	---	---	---	---	---
$20,000 - $49,999	−0.05	0.32	4.39	0.03	0.08
$50,000 and more	−0.42	2.10	2.90	0.06	0.23
R^2^	0.0308	0.0317	0.0192	0.0238	0.0903

Bold = statistical significance, *p < 0.05, **p < 0.01, ***p < 0.001, ref. = reference A1c = hemoglobin A1c, PCS = physical component of quality of life, MCS = mental component of quality of life adjusted for age, race, gender, site, diabetes duration, health status and comorbidity

**Table 5:** Adjusted models for the relationship with glycemic control and quality of life

	A1c	Blood Pressure	Lipids	PCS	MCS
Adjusted Analyses					
SAI score	0.001	0.33	0.60	−0.004	0.23**
R^2^	0.1058	0.0930	0.0259	0.0207	0.2004
SAI indicator - education					
No high school degree (ref)	---	---	---	---	---
High school graduate	−0.40	−2.36	4.00	−0.10	0.96**
College graduate	−0.37	−2.75	−1.70	0.03	0.58
Post college	−0.76*	0.92	12.90	0.03	0.85
SAI indicator - employment					
Unemployed (ref)	---	---	---	---	---
Retired	0.27	−0.78	−8.85	0.02	−0.13
Working part time	0.34	−0.03	−2.50	0.20	0.59
Working full time	0.71*	−0.02	−0.53	0.04	0.64*
SAI indicator – marital status					
Never married (ref)	---	---	---	---	---
Divorced/separated	0.17	2.00	−0.46	0.06	−0.37
Married	0.13	0.85	1.13	−0.17	−0.06
SAI indicator – substance abuse					
Abusing drugs and alcohol (ref)	---	---	---	---	---
Abusing drugs	−0.56	1.19	3.84	0.05	−0.41
Abusing alcohol	−0.62	−0.31	−4.02	−0.12	0.29
No drug or alcohol abuse	−0.39	1.04	−5.31	−0.18	0.31
SAI indicator – income					
> $20,000 (ref)	---	---	---	---	---
$20,000 - $49,999	0.07	1.91	7.23	0.05	0.06
$50,000 and more	−0.23	3.03	8.17	0.06	0.003
R^2^	0.1408	0.1038	0.0381	0.0397	0.2235

Bold = statistical significance, *p < 0.05, **p < 0.01, ***p < 0.001, ref. = reference A1c = hemoglobin A1c, PCS = physical component of quality of life, MCS = mental component of quality of life adjusted for age, race, gender, site, diabetes duration, health status and comorbidity

In the adjusted model (Table 5), the mental health component of the SF-12 was significantly associated with the SAI index (0.23, *p* < 0.01). When considering the individual SAI indicator variables, after adjustment for confounders, HbA1c still had a significant association with education (post-college education: beta −0.76, *p* < 0.05) and employment (working full-time: beta 0.71, *p* < 0.05). Additionally, the mental component of quality of life was associated with education (being a high school graduate: beta 0.96, *p* < 0.01) and employment (working full-time: beta 0.64, *p* < 0.05).

## Discussion

This study found that the relationship between the social adaptability index (SAI) score and self-care behaviors was not significant in adjusted models, and only showed significant association with the mental component of quality of life. When entering components of the SAI as individual variables, education and employment were significantly associated with HbA1c and the mental component of quality of life; education and income were associated with exercise; and substance abuse was associated with foot care. In this population of patients with type 2 diabetes, the SAI when used as a composite score has a low explanatory power and does not show the importance of individual factors or the differential relationship with diabetes self-care and outcomes. While SAI has been used successfully in the past, [13–16, 24–26] in this study, the index does not provide a good model fit and masks the relationship of individual components. While the use of a composite index to predict outcomes within a diabetes population would have high utility, particularly for clinical settings, this SAI lacked statistical and clinical significance in a representative diabetes population which suggests that five items alone are not adequate to explain or predict outcomes for patients with type 2 diabetes.

Prior research using the SAI has been done primarily using large cross-sectional national datasets among patients with chronic kidney disease [13–19]. One study considered a population with diabetes, however, in that study 85% of the sample had CKD of stage 2 or greater [16]. This is the first study to our knowledge to examine the relationship between SAI, self-care, and outcomes in a population with diabetes and more representative levels of comorbidities and severity. In this population, the results do not support SAI as a more specific or sensitive predictor in identifying populations at risk for poor health outcomes in diabetes. Instead, the results support a differential relationship between individual variables used in the SAI and diabetes self-care/outcomes. This supports the use of social determinant factors as a framework for prevention and treatment models for diabetes [6–8] and suggests consideration of these variables individually may be more worthwhile than use of index scores.

Research over the past decade has shown important effects of social determinants on both individual and population health, with more recent work being done on the direct effect social determinants has on adults with type 2 diabetes [7, 8]. The literature supports an association between a number of socioeconomic and psychosocial factors and diabetes outcomes such as income, depression and diabetes distress; all showing consistent associations with glycemic control and mortality [8, 9]. As such, a variety of determinants shown to be important in diabetes are not represented in the SAI index. In addition, three of the five SAI components are socioeconomic status based; however, the mechanism through which socioeconomic status influences diabetes related outcomes is not well understood [27]. For example, a recent study conducted a mediation analysis on eight possible pathways between education and diabetes incidence [28]. Pathways including depression, job control and health behaviors showed no mediation, with only BMI showing significant mediation in the analysis [28]. A separate path analysis investigating multiple socioeconomic variables found processes of care and access to care as mediators for income [8]. Therefore, even different socioeconomic factors may operate through different pathways, and a composite scale such as SAI may mask these relationships and make it difficult to develop effective interventions.

This study is strengthened by testing the social adaptability index in primary data in a population of adults with type 2 diabetes. However, there are several limitations worth mentioning. First, this study was conducted in the southeastern United States and as such may not be representative of the general US population. Second, the data used was cross-sectional and cannot speak to causality. Finally, instruments used in this study were validated scales, however, the data collected was based on self-report.

## Conclusion

In conclusion, while the social adaptability index has been used as a predictor of mortality and health outcomes in populations with chronic kidney disease, this study found that in populations with type 2 diabetes, SAI when used as a composite score, does not provide good explanatory power and masks the relationship of individual components that contribute to the understanding of self-care and health outcomes in diabetes. This study suggests the SAI indicator variables may provide better explanatory ability than the SAI composite score in adults with type 2 diabetes. However, additional studies in more diverse populations of adults with type 2 diabetes and populations with type 1 diabetes are needed to validate these findings. More importantly, additional studies on the predictive ability or explanatory power of the SAI composite score and indicator variables in other chronic diseases outside of chronic kidney disease are needed.

## Abbreviations

BMI: Body Mass Index
HbA1c: Hemoglobin A1c
MCS-12: Mental health
PCS-12: Physical health
SAI: Social Adaptability Index
SDSCA: Summary of diabetes self-care activities
